# Radioimmunotherapy in Non-Hodgkin’s Lymphoma: Retrospective Adverse Event Profiling of Zevalin and Bexxar

**DOI:** 10.3390/ph12040141

**Published:** 2019-09-20

**Authors:** Christos Sachpekidis, David B. Jackson, Theodoros G. Soldatos

**Affiliations:** 1 Department of Nuclear Medicine, Inselspital, Bern University Hospital, University of Bern, Bern 3010, Switzerland; christos_saxpe@yahoo.gr; 2 Molecular Health GmbH, Heidelberg 69115, Germany; jackson@molecularhealth.com

**Keywords:** non-Hodgkin’s lymphoma, radioimmunotherapy, ^90^Y-ibritumomab tiuxetan (Zevalin), ^131^I-tositumomab (Bexxar), FDA’s Adverse Event Reporting System (FAERS), VigiBase

## Abstract

The development of monoclonal antibodies has dramatically changed the outcome of patients with non-Hodgkin’s lymphoma (NHL), the most common hematological malignancy. However, despite the satisfying results of monoclonal antibody treatment, only few NHL patients are permanently cured with single-agent therapies. In this context, radioimmunotherapy, the administration of radionuclides conjugated to monoclonal antibodies, is aimed to augment the single-agent efficacy of immunotherapy in order to deliver targeted radiation to tumors, particularly CD20+ B-cell lymphomas. Based on evidence from several trials in NHL, the radiolabeled antibodies ^90^Y-ibritumomab tiuxetan (Zevalin, Spectrum Pharmaceuticals) and ^131^I-tositumomab (Bexxar, GlaxoSmithKline) received FDA approval in 2002 and 2003, respectively. However, none of the two radioimmunotherapeutic agents has been broadly applied in clinical practice. The main reason for the under-utilization of radioimmunotherapy includes economic and logistic considerations. However, concerns about potential side effects have also been raised. Driven by these developments, we performed retrospective analysis of adverse events reporting Zevalin or Bexxar, extracted from the FDA’s Adverse Event Reporting System (FAERS) and the World Health Organization’s VigiBase repository. Our results indicate that the two radioimmunotherapeutic agents have both related and distinct side effect profiles and confirm their known toxicological considerations. Our work also suggests that computational analysis of real-world post-marketing data can provide informative clinical insights. While more prospective studies are necessary to fully characterize the efficacy and safety of radioimmunotherapy, we expect that it has not yet reached its full therapeutic potential in modern hematological oncology.

## 1. Introduction

The development and introduction of monoclonal antibodies in clinical practice have constituted a true revolution in the management of non-Hodgkin’s lymphoma (NHL), the most frequent hematologic malignancy. *CD20*, a transmembrane calcium channel protein, represents the most common target antigen for monoclonal antibody therapy, since it is expressed at high density on most B-cell lymphomas [1]. Rituximab was the first monoclonal antibody approved by the U.S. Food and Drug Administration (FDA) in 1997, for the treatment of relapsed or refractory, low-grade (indolent) or follicular, CD20+ NHL. Rituximab is directed against the *CD20* antigen and exerts its antitumor effect by activation of antibody-dependent cell-mediated cytotoxicity through complement activation and induction of apoptosis [2]. However, despite the satisfying results of monoclonal antibody treatment, only few patients are permanently cured with single-agent therapy: fewer than half of follicular NHL patients respond to Rituximab with median response duration of about a year, since they may not respond or may develop resistance to antibody therapy [3,4].

Radioimmunotherapy represents an attempt to augment the single-agent efficacy of this therapeutic approach in NHL by conjugating therapeutic radionuclides to the monoclonal antibodies in order to deliver radiation to these tumors [5], which are known to be more radiosensitive than solid tumors and other types of cancer [6,7,8,9]. The mechanism of action of radioimmunotherapy on tumor cells involves a combination of the antibody-stimulated cytolysis and induction of apoptosis, with the ionizing radiation emitted from the radioisotope, which generates free radicals leading to cellular damage. Moreover, due to the “crossfire” radiation effect, adjacent tumor cells that do not bind the antibody may still be killed [5].

The radiolabeled antibodies ^90^Y-ibritumomab tiuxetan (Zevalin; Spectrum Pharmaceuticals) and ^131^I-tositumomab (Bexxar; GlaxoSmithKline) received FDA approval in 2002 and 2003, respectively, based on impressive results from several trials [10,11,12,13]. Zevalin was initially approved for treatment of patients with relapsed or refractory low-grade, follicular B-cell NHL, including patients with Rituximab-refractory follicular NHL. In 2014, it received expanded approval to include the first-line consolidation therapy of NHL patients following initial treatment with front-line chemotherapy; this approval was based on the results of a randomized multicenter phase III trial involving 414 patients with newly diagnosed advanced follicular lymphoma [14]. Bexxar was also approved for the treatment of relapsed, refractory, and transformed indolent lymphomas.

Despite the proven efficacy of radioimmunotherapy, not only in the relapsed/refractory setting but also in newly diagnosed patients with high response rates and durable remissions [14,15,16,17,18], this treatment modality has failed to be widely adopted by the hematooncology community. As a result, this led to commercial failure of the two products, reaching its nadir in 2014 with the discontinuation of U.S. production of Bexxar due to decreasing sales of the agent [4,19].

Although economic and logistic considerations have played a key role in the underuse of radioimmunotherapy, concerns about potential side effects related to radiation exposure, particularly the development of myelodysplastic syndrome (MDS) and acute myeloid leukemia (AML), have also been raised [4]. Driven by these developments, we sought to characterize the safety profile of this treatment modality based on accessible sources of real-world data. For this reason, adverse events (AEs) of patients receiving Zevalin or Bexxar reported in the FDA’s Adverse Event Reporting System (FAERS) and in the World Health Organization’s (WHO) global Individual Case Safety Report (ICSR) database (VigiBase) were extracted and analyzed.

## 2. Materials and Methods

We profiled Bexxar and Zevalin AEs by extracting data from the FAERS (incl. 2017Q2) and VigiBase (incl. 2018Q2) repositories.

### 2.1. Data Integration

The public FAERS dataset contained 7.9 million cases and held records regarding the patients’ treatments (medications), the indications of those treatments (disease or condition), and the observed reactions and outcomes (e.g., “death” or “hospitalization”) reported in these AEs. To compensate for ambiguities introduced by the non-standardized use of drug names [20], FAERS free-text medication descriptions were consolidated via a stepwise process that matched each name to standardized dictionaries [21]. Indications and reactions, coded by FAERS in terms from the Medical Dictionary for Regulatory Activities (MedDRA), were analyzed at the Preferred Term (PT) level (i.e., MedDRA Level 4 descriptions). Similarly, we processed the VigiBase dataset that held 17095324 AE cases.

### 2.2. Statistical Analysis

From each dataset (FAERS, VigiBase), we extracted two separate AE cohorts for Bexxar and Zevalin, respectively defined as follows:

- FAERS Bexxar cohort: 290 AEs (FAERS cases annotated with the ‘Tositumomab’ drug record).
- FAERS Zevalin cohort: 1196 AEs (FAERS cases annotated with the ‘Ibritumomab’ drug record).
- VigiBase Bexxar cohort: 307 AEs (VigiBase cases with product (drug) names mentioning ‘Bexxar’ or ‘Tositumomab’).
- VigiBase Zevalin cohort: 1125 AEs (VigiBase cases with product (drug) names mentioning ‘Zevalin’ or ‘Ibritumomab’).

For the statistical characterization of the cohorts, we employed the proportional reporting ratio (PRR) metric, an established measure of disproportionality in pharmacovigilance for the evaluation of signals generated from spontaneous reporting data [22]. PRRs were computed using the approach described by van Puijenbroek et al. [23].

Each cohort was characterized with respect to the occurrence of drugs, outcomes, indications and reactions reported in the respective AEs. We also considered relevant statistical significance reflected by Fisher’s exact test *p*-values (two-tailed). In this study, we considered statistical significance indicated by a *p*-value of 5% or lower (i.e., when *p*-value < 0.05). To structure the statistical analysis, each cohort’s AEs were compared against the remaining respective dataset’s AEs (whether FAERS or VigiBase) during the calculation of PRR and Fisher’s exact test scores.

Results for each cohort are summarized in the Supplementary Material file: results per cohort list the observed case counts (i.e., number of AEs that a certain occurrence was observed in each cohort), percentage of occurrence within each cohort’s cases, and the respective PRR disproportionality scores and Fisher’s test *p*-values. The Supplementary Material file contains all observations for consideration by the community, irrespective of their PRR or Fisher’s exact test signals.

## 3. Results

Our dataset consisted of AEs extracted from FAERS and VigiBase that reported use of Bexxar or Zevalin. As expected, ‘non-Hodgkin’s lymphoma’, as well as other PTs describing—more or less specific—forms of lymphoma were reported as treatment indication in the vast majority of the reported cases in all cohorts (see Supplementary Material file). With the exception of three patient cases, where both drugs have been co-reported—most likely in succession one after the other—Zevalin and Bexxar were not reported in the same AEs. The most frequent medication reported with Zevalin was Rituximab, mentioned in 70% of its FAERS AEs. In comparison, Rituximab was mentioned only in 15% of Bexxar’s AEs in FAERS.

**Figure 1:**
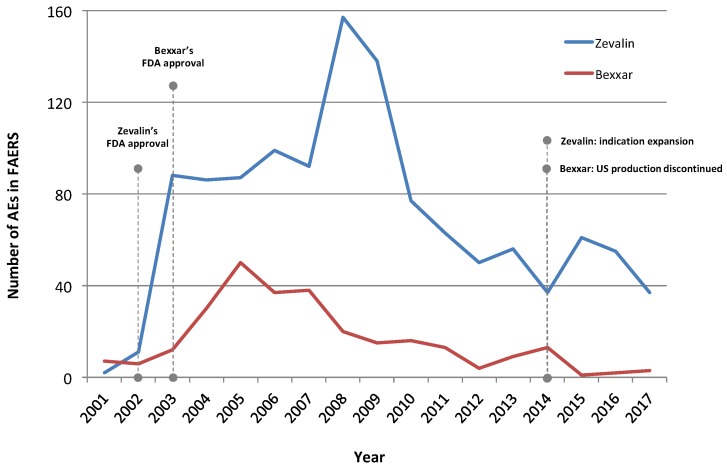
Timeline of AEs reporting Zevalin and Bexxar in the FDA’s Adverse Event Reporting System (FAERS). Overall, there are more AEs identified for Zevalin in FAERS, likely indicating broader usage adoption than for Bexxar. Fourteen cases reporting Bexxar prior to 2001 are not displayed. Also, the distribution of AEs over time does not include incompletely reported cases for which no date was specifically registered. Last, AEs dated prior to a drug’s approval may reflect reports from preapproval studies and clinical trials. The reduction of AE numbers for 2017 is explained by the fact that the full dataset for that year was not yet released by FAERS at the time this analysis took place. Note that a certain time delay between the disclosure of reports to FAERS and the date of an event or the use of an agent is reasonable to be expected.

The identified AE trends were likely reflective of clinical usage trends (Figure 1), including the decreasing course of their use during the last years. Despite the certain collection lag existing in the capture of respective AEs, the historical timeline portrays both the initial increase that followed the agents’ FDA approvals as well as their decline, particularly for Bexxar. Zevalin’s temporary increase after 2014 probably represents its indication expansion to include the first-line consolidation therapy of NHL patients following initial treatment with front-line chemotherapy.

Overall, FAERS and VigiBase contained similar adverse reaction profiles for each radioimmunotherapeutic agent. Table 1 juxtaposes key patient outcomes reported in the respective radioimmunotherapeutic agent cohorts.

### 3.1. Adverse Event Profiling

Next, side effect profiles for those cohorts were extracted: Table 2, Table 3, Table 4 and Table 5 summarize up to the thirty most frequently reported reactions in each cohort, described in MedDRA PTs (i.e., level 4 terms). The following PTs did not reflect drug-induced effects and were therefore excluded from these tables, whenever mentioned: ‘Disease progression’, ‘Malignant neoplasm progression’, ‘Drug ineffective’, ‘Stem cell transplant’, ‘Non-Hodgkin’s lymphoma’, ‘Death’.

#### 3.1.1. Zevalin Reactions

In both datasets (FAERS, VigiBase), cytopenias were the most commonly reported reactions with Zevalin (Table 2 and Table 3). More specifically, ‘thrombocytopenia’, and ‘neutropenia’ were the most frequent reaction PTs, followed by ‘leukopenia’ and ‘pancytopenia’. Among the most frequently reported reaction PTs there were only few infusion related terms (such as urticaria, hypotension, angioedema, hypoxia, bronchospasm, or cardiogenic shock), with the exception of ‘dyspnea’ that was reported in 2.1% of the VigiBase Zevalin cohort. Reaction PT ‘pyrexia’ had a somewhat increased PRR (3.4 in FAERS, 1.4 in VigiBase) and even though it might represent an infusion related reaction, it could also occur in the context of an infection or inflammation. ‘Sepsis’, ‘febrile neutropenia’ and a variety of infections and inflammations (including ‘mucosal inflammation’) were also often reported with the agent and had increased PRR scores. Interestingly, reaction PT ‘Meningitis tuberculous’ had a very high PRR signal in both Zevalin datasets. Among the reaction PTs with high PRR signals were also ‘Myelodysplastic syndrome’ (MDS) and ‘acute myeloid leukemia’ (AML). MDS was reported in 6.9% and 6.1% of the corresponding FAERS and VigiBase cohorts, while AML was reported in 3.6% and 2.9% of the respective FAERS and VigiBase Zevalin cohorts.

#### 3.1.2. Bexxar Reactions

Bexxar cohorts were smaller in size as compared to the Zevalin ones. The most frequently reported PTs with Bexxar were MDS (15.9% in FAERS, 14.0% in VigiBase), ‘pyrexia’ (10.3% in FAERS, 8.8% in VigiBase), AML (7.6% in FAERS, 8.8% in VigiBase), ‘fatigue’ (7.6% in FAERS, 7.2% in VigiBase) and ‘dyspnea’ (7.2% in FAERS, 6.5% in VigiBase). As in the case of Zevalin, cytopenias were also frequently reported with Bexxar in both datasets. In contrast, allergic/infusion reaction PTs such as ‘dyspnea’, ‘hypotension’, ‘chills’ and ‘vomiting’ were recorded in a higher proportion of the respective cohorts for Bexxar (Table 4 and Table 5).

## 4. Discussion

Despite the promising results from clinical trials providing evidence regarding the efficacy and safety of radioimmunotherapy, neither of the two approved radioimmunotherapeutic agents found broad application in clinical practice. Bexxar exited the US market in 2014 after low sales, whereas nowadays Zevalin is not often used in the treatment of NHL. Although economic and logistic considerations played an important role in the underutilization of these agents [24], one other key concern regarding radioimmunotherapy is its toxicity profile and, more specifically, bone marrow damage and unexpected late side effects—particularly, the development of MDS and AML [3,25].

In this work, we examined such considerations by analyzing real world post-marketing AE data extracted from FAERS and VigiBase. Our results provide additional insights into the safety profile of radioimmunotherapy. Table 6 summarizes the most frequently reported reactions for Zevalin and Bexxar in both datasets by organizing them in groups of main classes.

The analysis of the Zevalin AE cohorts confirmed its known toxicological profile [26,27,28]. The high occurrence of cytopenias may be attributed to bone marrow suppression induced by the agent, while infections, inflammations and related side effects were also often reported, with sepsis, pyrexia and febrile neutropenia being the most frequent ones. Despite its consideration as one of the most common Zevalin AEs, ‘nasopharyngitis’ was only occasionally reported as such (mentioned only in two AE cases of the FAERS Zevalin cohort, as reaction) [26]. Interestingly, ‘nausea’, ‘dyspnea’ and ‘vomiting’ had lower PRR scores (<1), despite their reported frequency in the FAERS and/or VigiBase Zevalin cohorts (>2%). In contrast, the data suggest ‘tuberculous meningitis’ as a rather specific reaction PT related to the Zevalin cohorts, containing a significant 17.5% and 14.8% of the term’s total mentioning in FAERS and VigiBase, respectively. However, we could not confirm this observation in the literature, nor assess further whether this occurrence could be attributed to some sort of reporting bias or co-medication effects. Without access to detailed clinical data or the original AE narratives, we observed that almost all of Zevalin’s FAERS tuberculous meningitis AEs were in patients being pre- or co-medicated with chemotherapeutic agents such as Vincristine, Doxorubicin and Etoposide, as well as Prednisone.

Secondary malignancies, namely MDS and AML, were also reported with Zevalin. More specifically, MDS was reported with Zevalin in FAERS with an occurrence of 6.9% and AML with 3.6%, and respectively in 6.1% and 2.9% of the VigiBase Zevalin cohort. This is in agreement with the cumulative incidence of MDL/AML in 5.2% of patients with relapsed or refractory NHL enrolled in Zevalin clinical studies [26]. However, the majority of Zevalin patients first receive Rituximab treatment and it is therefore difficult to determine which of these agents has primarily contributed to the occurrence of MDS and AML in the examined AEs, especially when Rituximab itself is also considered as a potential risk factor for the development of these secondary malignancies [29,30,31,32]. One other observation that supports this concern is the reporting of ‘progressive multifocal leukoencephalopathy’ and ‘cytomegalovirus infection’, which are identified risks of Rituximab therapy [29]. Finally, ‘mucosal inflammation’—a significant consideration in terms of Zevalin treatment—was reported in 2.7% of the FAERS Zevalin cohort, whereas only few serious infusion related reactions, which are considered potentially severe during therapy with Zevalin and sometimes even fatal [26], were reported.

The analysis of the smaller Bexxar cohorts revealed a somewhat different toxicological profile, in comparison to Zevalin. This is in part expected, given that ibritumomab tiuxetan is radiolabeled with ^90^Y while tositumomab is radiolabeled with ^131^I—even though both radionuclides are beta emitters, they possess different characteristics regarding physical half-life, maximum beta energy, penetration length of their beta emissions, as well as emission of gamma radiation. Such physical differences of the radionuclides directly influence both the biodistribution and the dosimetry of the radioimmunoconjugate applied, which in turn affect their toxicity. To determine radiation exposure to various organs and to confirm tumor targeting, dosimetry is incorporated into the design of radioimmunotherapy regimens [33]. However, dosimetry is not required with Zevalin, since the first dosimetry analyses of the agent in several clinical trials with relapsed or refractory NHL showed that radiation absorbed doses were within safety limits and did not correlate with toxicity [34,35]. While the administered activity of Zevalin is mainly determined by patient weight and baseline platelet count, dosimetry before radioimmunotherapy with the ^131^I-labeled Bexxar is mandatory to determine the appropriate delivered activity [36].

The first difference between the two agents related to the occurrence of secondary malignancies that were reported in a higher proportion among Bexxar’s cohorts: MDS was reported in 15.9% and AML in 7.6% of the respective FAERS cohort and, accordingly, in 14.0% and 8.8% of the VigiBase cohort. These findings are in line with the cumulative incidence of MDS/secondary leukemia observed in 10% of patients enrolled in Bexxar clinical trials with a median follow-up of 39 months [37]. Infusion/allergic reactions were also reported in higher proportions among Bexxar AEs as compared to Zevalin, with ‘dyspnea’, ‘hypotension’, ‘chills’, ‘vomiting’ and ‘rash’ being mentioned in more than 3% of each cohort’s cases. However, ‘nausea’, ‘rash’ and ‘vomiting’ had lower PRR scores (≤1) in Bexxar’s VigiBase cohort. Moreover, pyrexia, which could be either an infusion reaction or an infection sign, was in both Bexxar datasets second only to MDS and AML.

Interestingly, cytopenias were reported in smaller proportions of the Bexxar cohorts, when compared to Zevalin. Similarly, this was the case also for infections, inflammations and their related reactions, with pyrexia, febrile neutropenia and pneumonia being the most frequently reported. Hypothyroidism, a main concern regarding Bexxar usage due to Iodine ^131^I, was only occasionally reported (mentioned only in one AE case of the VigiBase Bexxar cohort, as reaction). One possible reason for this might be the effective use by the community of thyroid-blocking medication prior to the administration of this radioimmunotherapeutic regimen.

Overall, results from the analysis of Zevalin AEs confirmed its known toxicological profile, consistent with findings from clinical trials as well as descriptions presented in the agent’s label. However, among Bexxar AEs secondary malignancies ranked first, before other commonly expected side effects such as cytopenias, infections, infusion reactions, asthenia or nausea, observed in > 25% of treated patients in some clinical trials [37]. This may be explained by the fact that FAERS and VigiBase contain reports of AEs and not all potential side effects, as documented in clinical trials and prospective studies. Bexxar cohorts were also smaller in size compared to Zevalin, therefore possibly not equivalently representative. One other aspect to consider is the potential bias that may underlie radioimmunotherapy AE reporting. Such a bias may be manifested in several ways, such as the use of terms coming from a particular terminology (medical/technical language), reporting of AEs occurring/examined in specific circumstances, prejudice of reporters (e.g., affected by media or peer commentaries), as well as failure to mention some reactions simply because they are known or not severe. Reporting bias may, for example, explain over- or under-representation of certain effects, such as in the cases of nasopharyngitis, or the reporting of infusion reactions among Zevalin AEs. Furthermore, causal etiologies underlying the observed events could not be clearly determined as both Zevalin and Bexxar were frequently mentioned with agents known to potentially increase the risk or severity of adverse effects when combined together (such as Rituximab, Cyclophosphamide, Prednisone, Vincristine, Doxorubicin, Fludarabine and Etoposide).

Due to practical limitations, the present study could not consider specific severity grading of reported reactions, information regarding treatment duration or previous therapies, de-/re-challenge information, patient/event history and demographics, as well as data regarding dosage of the applied radioactive pharmaceuticals. Moreover, reported frequencies do not represent occurrence in the general treated population, but in patients who manifested AEs under therapy and their incidence was reported. As with other studies of AEs e.g., [38,39], our results should therefore not be interpreted as calculated incidences of side effects in the general NHL patient population receiving these radioimmunotherapeutic agents. This is not the intended use of data coming from spontaneous AE repositories since they contain only AEs and are therefore biased without proper normalization considering reference/control data.

Nonetheless, AE analytics provide certain advantages and systematic insights in the toxicological profiling of therapeutic agents. Not only do they serve as an augmented data stream capturing real-world scenarios regarding therapeutic uses and conditions not studied in clinical-trials, but they also include information for many more patients [21,40]. When available, AE analytics can further benefit from the examination of additional data regarding laboratory and clinical parameters. For example, molecular dissection of AEs has been shown to effectively improve clinical insights, as well as inform about potential drug-drug interactions or other mechanisms underlying observed outcome phenotypes e.g., [41,42]. Such information could be important within the broader context of immunotherapy, potentially providing useful markers for the use of these agents in NHL patients. Indeed, results from recent NHL trial studies provide updated evidence regarding the efficacy and safety of radioimmunotherapy, possibly inspiring reconsideration of Zevalin and Bexxar’s clinical utility in modern hematological oncology [43,44,45].

## 5. Conclusions

We investigated the toxicological profile of radioimmunotherapeutic agents on NHL, by performing a retrospective analysis of AE cases of patients receiving Zevalin or Bexxar, reported in FDA’s FAERS and WHO’s VigiBase repositories. Overall, our results recapitulate known side effects observed with radioimmunotherapy and confirm cytopenias, infections, inflammations, infusion reactions, as well as MDS and AML as some of the main patient safety concerns. The present work also suggests that computational analysis of real-world patient outcome data can provide informative insights in the field of the toxicological profiling of therapeutic agents. While such analytics can certainly benefit from the consideration of additional clinical information, we also call for more studies required to characterize the efficacy and safety of radioimmunotherapy in NHL.

## Figures and Tables

**Table 1:** Selected outcomes reported in the two datasets; all adverse event (AE) cases considered per cohort. Note that the outcomes associated to a single event may be more than one.

Outcome	% Zevalin AEs		% Bexxar AEs	
	FAERS	VigiBase	FAERS	VigiBase
Death	33.4	22.8	22.1	24.1
Hospitalization	41.5	22.1	31.0	20.5
Life threatening	7.4	3.9	7.9	5.2
Other	46.3	31.8	36.6	23.1

**Table 2:** Most frequently reported MedDRA preferred term (PT) reactions (level 4 terms) with Zevalin in FAERS. All observations are statistically significant, with *p*-value < 0.05.

Reaction (MedDRA PT) Name	Number of AEs_in Cohort	PRR	Cohort %
Thrombocytopenia ^1^	151	21.4	12.6
Neutropenia	124	19.0	10.4
Sepsis	98	13.0	8.2
White blood cell count decreased ^2^	84	13.2	7.0
Myelodysplastic syndrome (MDS)	83	74.1	6.9
Platelet count decreased ^1^	80	12.0	6.7
Pyrexia	79	3.4	6.6
Pancytopenia	78	20.0	6.5
Febrile neutropenia	76	20.9	6.4
Anaemia ^3^	65	4.7	5.4
Bone marrow failure	63	37.3	5.3
Diarrhoea	60	1.7	5.0
Pneumonia	57	2.9	4.8
Haemoglobin decreased ^3^	51	6.6	4.3
Meningitis tuberculous	49	1489.1	4.1
Multi-organ failure	44	15.9	3.7
Acute myeloid leukaemia (AML)	43	42.5	3.6
Leukopenia ^2^	43	13.6	3.6
Neutrophil count decreased	42	19.4	3.5
Progressive multifocal leukoencephalopathy	38	64.5	3.2
Nausea	36	0.7	3.0
Cytomegalovirus infection	33	37.4	2.8
Septic shock	33	12.7	2.8
Mucosal inflammation	32	19.7	2.7
Infection	32	4.0	2.7
Renal failure	31	3.5	2.6

^1^ The terms ‘Thrombocytopenia’ and ‘Platelet count decreased’ refer to the same condition. ^2^ The terms ‘White blood count decreased’ and ‘Leukopenia’ refer to the same condition. ^3^ The terms ‘Anaemia’ and ‘Haemoglobin decreased’ refer to the same condition.

**Table 3:** Most frequently reported MedDRA PT reactions (level 4 terms) with Zevalin in VigiBase. All observations are statistically significant, with *p*-value < 0.05.

Reaction (MedDRA PT) Name	Number of AEs_in Cohort	PRR	Cohort %
Thrombocytopenia ^1^	165	20.1	14.7
Neutropenia	157	24.2	14.0
Platelet count decreased ^1^	117	35.2	10.4
Pancytopenia	90	37.2	8.0
Sepsis	71	20.0	6.3
White blood cell count decreased ^2^	71	18.4	6.3
Myelodysplastic syndrome (MDS)	69	170.1	6.1
Anaemia ^3^	59	6.1	5.2
Pneumonia	53	5.2	4.7
Pyrexia	51	1.4	4.5
Febrile neutropenia	50	26.2	4.4
Leukopenia ^2^	48	9.1	4.3
Neutrophil count decreased	42	24.1	3.7
Meningitis tuberculous	37	3877.3	3.3
Nausea	37	0.6	3.3
Fatigue	34	1.2	3.0
Diarrhoea	34	1.0	3.0
Acute myeloid leukaemia (AML)	33	93.7	2.9
Bone marrow failure	32	15.1	2.8
Pain	29	1.2	2.6
Progressive multifocal leukoencephalopathy	27	131.5	2.4
Haemoglobin decreased ^3^	25	7.0	2.2
Dyspnoea	24	0.7	2.1
Dehydration	23	4.6	2.0
Vomiting	23	0.6	2.0
Asthenia	23	1.2	2.0

^1^ The terms ‘Thrombocytopenia’ and ‘Platelet count decreased’ refer to the same condition. ^2^ The terms ‘White blood count decreased’ and ‘Leukopenia’ refer to the same condition. ^3^ The terms ‘Anaemia’ and ‘Haemoglobin decreased’ refer to the same condition.

**Table 4:** Most frequently reported MedDRA PT reactions (level 4 terms) with Bexxar in FAERS. All observations are statistically significant, with *p*-value < 0.05.

Reaction (MedDRA PT) Name	Number of AEs_in Cohort	PRR	Cohort %
Myelodysplastic syndrome (MDS)	46	168.7	15.9
Pyrexia	30	5.3	10.3
Acute myeloid leukaemia (AML)	22	89.4	7.6
Fatigue	22	2.0	7.6
Dyspnoea	21	2.4	7.2
Nausea	18	1.4	6.2
Anaemia	17	5.1	5.9
Thrombocytopenia ^+^	16	9.3	5.5
Pancytopenia	14	14.8	4.8
Hypotension	14	4.4	4.8
Febrile neutropenia	13	14.7	4.5
Neutropenia	13	8.2	4.5
Chills	13	6.7	4.5
Lymphadenopathy	12	20.4	4.1
Pneumonia	12	2.6	4.1
Vomiting	12	1.6	4.1
Dizziness	12	1.5	4.1
Infusion related reaction	11	14.9	3.8
Rash	11	1.9	3.8
Platelet count decreased ^+^	10	6.2	3.4
Back pain	10	2.7	3.4
Arthralgia	10	1.8	3.4
Asthenia	10	1.7	3.4
Pleural effusion	9	8.1	3.1
Cardiac failure congestive	9	4.2	3.1
Decreased appetite	9	2.5	3.1
Weight decreased	9	2.1	3.1

^+^ The terms ‘Thrombocytopenia’ and ‘Platelet count decreased’ refer to the same condition.

**Table 5:** Most frequently reported MedDRA PT reactions (level 4 terms) with Bexxar in VigiBase. All observations are statistically significant, with *p*-value < 0.05.

Reaction (MedDRA PT) Name	Number of AEs_in Cohort	PRR	Cohort %
Myelodysplastic syndrome (MDS)	43	386.9	14.0
Acute myeloid leukaemia (AML)	27	280.5	8.8
Pyrexia	27	2.7	8.8
Fatigue	22	2.8	7.2
Dyspnoea	20	2.3	6.5
Thrombocytopenia ^+^	18	8.0	5.9
Nausea	17	1.0	5.5
Febrile neutropenia	15	28.7	4.9
Pancytopenia	15	22.7	4.9
Anaemia	15	5.7	4.9
Neutropenia	13	7.3	4.2
Pneumonia	13	4.7	4.2
Hypotension	13	4.0	4.2
Chills	13	3.7	4.2
Lymphadenopathy	12	16.6	3.9
Infusion related reaction	11	24.8	3.6
Infection	11	8.3	3.6
Back pain	11	4.1	3.6
Rash	11	0.8	3.6
Platelet count decreased ^+^	10	11.0	3.3
Pain in extremity	10	2.8	3.3
Arthralgia	10	2.2	3.3
Asthenia	10	1.9	3.3
Vomiting	10	0.9	3.3
White blood cell count decreased	9	8.6	2.9
Cardiac failure congestive	9	7.9	2.9
Weight decreased	9	3.7	2.9

^+^ The terms ‘Thrombocytopenia’ and ‘Platelet count decreased’ refer to the same condition.

**Table 6:** Most frequently reported MedDRA PT reactions (level 4 terms) with Zevalin and Bexxar in both datasets categorized in groups.

Zevalin	Bexxar
**Blood/Cytopenias**	
Thrombocytopenia Neutropenia Leukopenia Pancytopenia Anemia Bone marrow failure	Anemia Thrombocytopenia Pancytopenia Neutropenia
**Infections/Inflammations**	
Sepsis Pyrexia ^+^ Febrile neutropenia Pneumonia Meningitis tuberculous Cytomegalovirus infection Septic schock Mucosal inflammation	Pyrexia ^+^ Febrile neutropenia Chills Pneumonia
**Secondary malignancies**	
Myelodysplastic syndrome (MDS)	Myelodysplastic syndrome (MDS)
Acute myeloid leukaemia (AML)	Acute myeloid leukaemia (AML)
**Gastrointestinal**	
Diarrhoea Nausea	Nausea
**Other**	
Multi-organ failure Progressive multifocal leukoencephalopathy Renal failure Fatigue Pain Asthenia Dehydration	Fatigue Dyspnoea Hypotension Chills Lymphadenopathy Dizziness Infusion related reaction Rash Back pain Arthralgia Asthenia Pleural effusion Cardiac failure congestive Decreased appetite Weight decreased

^+^ The term ‘pyrexia’ could be an infusion reaction but also an infection sign.

## Supplementary Materials

The following are available online at https://www.mdpi.com/1424-8247/12/4/141/s1.
